# An Herbal Formula CG^plus^ Ameliorates Stress-Induced Hepatic Injury in a BALB/c Mouse Model

**DOI:** 10.3389/fphar.2020.00447

**Published:** 2020-04-14

**Authors:** Hyeong-Geug Kim, Yun-Hee Kim, Sung-Bae Lee, Jin-Seok Lee, Sung-Wook Chae, Dong-Gu Kim, Chang-Gue Son

**Affiliations:** ^1^ Liver and Immunology Research Center, Dunsan Oriental Hospital of Daejeon University, Daejeon, South Korea; ^2^ Korean Medicine Convergence Research Division, Korea Institute of Oriental Medicine (KIOM), Daejeon, South Korea; ^3^ Hanbang Cardio-Renal Syndrome Research Center, Wonkwang University, Iksan, South Korea

**Keywords:** stress, HPA axis, liver injury, oxidative stress, IL-1β

## Abstract

**Introduction:**

Stress is a well-known factor for inflammation in diverse organs/tissues. Stress also leads to liver injury, which was supported by clinical observations and animal studies. We herein investigated the hepatoprotective property of an herbal formula (called as CG^plus^) consisting of Artemisia gmelinii Weber ex Stechm. (syn, *Artemisia iwayomogi* Kitamura), Wurfbainia villosa var. xanthioides (Wall. ex Baker) Skornick. & A.D.Poulsen (syn, *Amomum xanthioides* Wallich), and *Salvia miltiorrhiza* Bunge against stress-induced hepatic damage.

**Methods:**

Male BALB/c mice were orally administered water extract of CG^plus^ (0, 50, 100, or 200 mg/kg) daily for 5 days, and then subjected to immobilization stress for 6 h on the 5^th^ day.

**Results:**

Acute immobilization stress elevated remarkably serum concentrations of stress hormones (corticosterone and adrenaline) and two hepatic injury parameters (ALT and AST), while these alterations were significantly attenuated by the administration of CG^plus^. The increases of oxidative parameters (ROS, NO, lipid peroxidation, and protein carbonyl) and deviation of IL-1β and IL-10 in opposite directions in hepatic tissues were significantly normalized by CG^plus^. Pre-treatment with CG^plus^ also notably ameliorated the abnormal activation of toll-like receptor 4 (TLR4), CD14, and lipopolysaccharide-binding protein (LPB) as well as infiltration of neutrophils in hepatic tissues.

**Conclusion:**

These results suggest that an herbal formula (CG^plus^) derived from traditional pharmaceutical theory has a potent protective effect against stress-induced hepatic injury *via* regulation of pro- (IL-1β) and anti-inflammatory (IL-10) cytokines.

## Introduction

Stress is an unavoidable part of human life, and it has been emphasized as a main cause of disease in traditional Oriental medicine. Individual responses to stressors *via* the activation of the hypothalamic-pituitary-adrenal (HPA) axis lead to the release of key peripheral mediators, such as glucocorticoids and catecholamines (Stratakis and Chrousos, 1995; Bugajski, 1999). However, uncontrolled stress has deleterious effects on the immune, cardiovascular, neuroendocrine, and central nervous systems and even cancer-related pathology (Schneiderman et al., 2005; Moreno-Smith et al., 2010).

Clinical observations have reported that psychosocial stress affects the clinical symptoms of hepatic disorders and hepatic chemistries (Fukudo et al., 1989; Kunkel et al., 2000). Several animal models have shown that stress aggravates toxic agent-induced liver damage and triggers liver injury in normal rodents (Fernández et al., 2000; Chida et al., 2004b). The corresponding mechanisms are thought to involve the alteration of hepatic blood flow (Chida et al., 2005), over productions of reactive oxygen species (ROS) (Kovács et al., 1996) and pro-inflammatory cytokines (Zhou et al., 2001) and gut-derived lipopolysaccharides (LPS) influx under stress condition (Lambert, 2009). However, the detail mechanisms are still unclear and any therapeutics does not exist yet.

On the other hand, liver disease is one of main death cause as accounting for approximately 2 million deaths per year worldwide (WHO, 2016). Based on accumulated scientific evidences, herbal plants or their active compounds have been successful in development of hepatotherapeutics such as silymarin/silybin (Federico et al., 2017). Based on the traditional use and experimental results, we formulated an herbal combination of Artemisia gmelinii Weber ex Stechm. (syn, *Artemisia iwayomogi* Kitamura), Wurfbainia villosa var. xanthioides (Wall. ex Baker) Skornick. & A.D.Poulsen (syn, *Amomum xanthioides* Wallich), and *Salvia miltiorrhiza* Bunge, called as CG^plus^ (**Supplementary Table 1**). This formula evidenced the hepatoprotective and anti-hepatofibrotic effects in dimethylnitrosamine-induced chronic hepatic injury rat model and non-alcoholic steatohepatitis mouse model (Kim et al., 2016; Lee et al., 2018). Those three medicinal plants also have been proved to have the hepatoprotective effects or antioxidative property in animal-based experiments (Han et al., 2012; Kim et al., 2014; Kim et al., 2015).

Based on above facts, we hypothesized that CG^plus^ can be a potential candidate modulating stress-derived hepatotoxicity. We herein adopted a mouse model of immobilization which has been adapted as a physical and psychological severe stress (Liu et al., 1994). And then we investigated the hepatoprotective effects of CG^plus^ and its underlying corresponding mechanisms on stress-related hepatic damage.

## Materials and Methods

### Composition of Formula (CG^plus^) and Standardization

**Figure 1:**
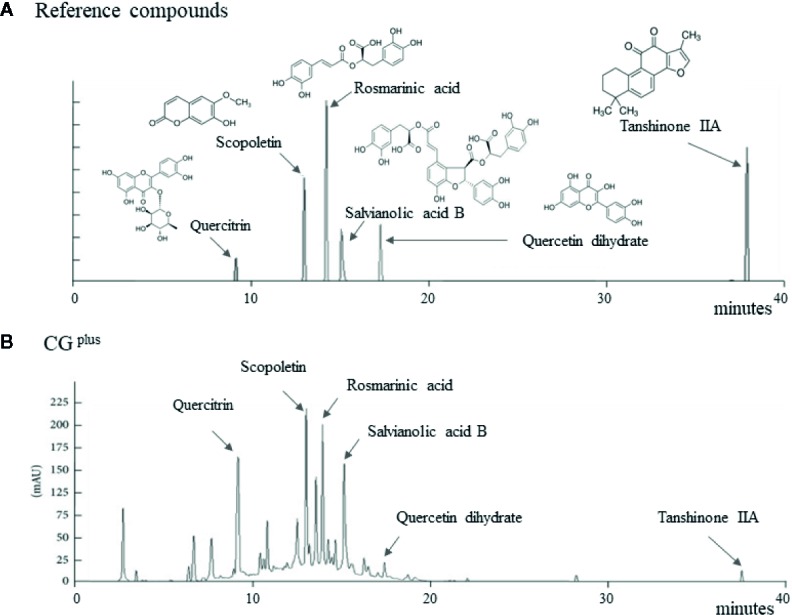
Fingerprint of CG^plus^. Chemical composition analysis of CG^plus^ was conducted using high-performance liquid chromatography (HPLC). Chromatogram of six reference compounds **(A)** and CG^plus^ **(B)** were shown.

*Salvia miltiorrhiza* Bunge, Artemisia gmelinii Weber ex Stechm. (syn, *Artemisia iwayomogi* Kitamura), and Wurfbainia villosa var. xanthioides (Wall. ex Baker) Skornick. & A.D.Poulsen (syn, *Amomum xanthioides* Wallich) were obtained from the Jeong-Seong Pharmaceutical Compay (Daejeon, Republic of Korea). All herbs were approved by Ministry of Food and Drug Safety (MFDS) in Korea, and the voucher specimen has been deposited at Jeong-Seong Pharmaceutical Company. The water-soluble extraction and fingerprinting were conducted as follows. Briefly, 100 g each of the three fully dried herbs were mixed and boiled separately in distilled water (DW) for 90 min and concentrated for 120 min. After filtration and lyophilization, the extract was stored at −70°C until use in Liver & Immunology Research Center, Daejeon Oriental Hospital of Daejeon University (Storage specimen # LIRC 2016-03). The final yield of the water extraction was 9.58%. Using an 1100 series high-performance liquid chromatography system (HPLC; Agilent Technologies, Santa Clara, CA), CG^plus^ fingerprint was conducted, along with the reference compounds, including scopoletin (in A. gmelinii), quercitrin and quercetin dehydrate (in W. villosa var. xanthioides), and rosmarinic acid, salvianolic acid B, and tanshinone IIA (in *S. miltiorrhiza*), respectively (**Figure 1**).

### Animals and Experimental Design

A total of 48 specific pathogen-free male BALB/c mice (6 weeks old, 19–21 g) were purchased from a commercial animal breeder (Daehan-Biolink, Chonng-buk,South Korea) and acclimated for 1 week before use. The animals were housed in an environmentally controlled room at 22 ± 2°C under a 12/12-h light/dark cycle. They were fed a commercial standard chow diet (AgriFurina; Cargill, Gyeonggido, Republic of Korea) with tap water *ad libitum*. The mice were randomly divided into six groups (*n* = 8). All mice were orally administered DW (naïve and control group), CG^plus^ (50, 100, or 200 mg/kg), or dimethyl dimethoxy biphenyl dicarboxylate (DDB; 50 mg/kg) using oral gavage once daily for 5 days. In addition, another experiment (total 12 mice, each 3 mice of four groups for naïve, control, CGplus 200 mg/kg and DDB mg/kg) was set for immunohistochemistry. DDB was adopted as positive compound based on its well-known hepatoprotective effects. Two hours after the last administration, mice (except naïve group) were subjected to immobilization stress by tying all four limbs tightly to a grid using quartz-pasted tape as previously described for 6 h (Kumar et al., 2010). Under ether anesthesia, all mice were sacrificed by complete blood collection from the abdominal vein. The liver tissues were immediately removed and stored at −70°C. Animal experiments were conducted in accordance with the rules for the Use of Laboratory Animals and directed by the United States National Institutes of Health as well as ARRIVE guidelines respectively. The protocol was approved by the Institutional Animal Care and Use Committee of Daejeon University (DJUARB2016-004).

### Analysis of Serum Stress Hormones and Biochemistry

Serum samples were obtained for subsequent separation after 1 h of blood clotting and centrifugation (3,000 × g, 15 min). The serum concentrations of stress-related hormones (corticosterone and adrenaline) were measured using commercial enzyme-linked immunosorbent assay kits according to the manufacturer’s protocols (LDN, Nordhorn, Germany). The serum levels of liver enzymes such as aspartate aminotransferase (AST) and alanine aminotransferase (ALT) were determined using an Auto Chemistry Analyzer (AU400; Olympus, Tokyo, Japan).

### Analysis of ROS and NO in the Hepatic Tissues

The total ROS levels in hepatic tissue were determined as described previously (Hayashi et al., 2007). Briefly, 100 mg hepatic tissue was homogenized in 1 ml of radioimmunoprecipitation assay (RIPA) buffer. Then, 5 µl of sample was added to 140 µl of 0.1 M sodium acetate buffer (pH 4.8) in 96-well micro-plate. After incubation at 37°C for 5 min, 100 µl of the N, N-diethyl-pera-phenylenediamine (DEPPD) and ferrous sulfate combined mixture solutions were added to the each sample. The products were measured at 505 nm absorbance using a micro-plate reader (Molecular Device Corp., Sunnyvale, CA, USA).

Nitro oxide (NO) levels in hepatic tissues were determined using the Griess method (Green et al., 1982). Briefly, 40 μl homogenate was mixed with 160 μl Griess reagent (1% sulfanilamide, 0.1% N-(1-naphthyl) ethylenediamine hydrochloride, and 2.5% H_3_PO_4_) in a 96-well plate. After incubation at 37°C for 20 min, the purple azo dye product was measured at 540 nm using UV spectrophotometer.

### Analysis of Lipid Peroxidation and Protein Carbonyl in the Hepatic Tissues

The hepatic levels of malondialdehyde (MDA), as a representative lipid peroxidation product, were measured using the thiobarbituric acid reactive substances (TBARS) method, as described previously (Uchiyama and Mihara, 1978). Briefly, 200 mg of liver tissue homogenized in ice-cold KCl (1.15%) was mixed with 1% H3PO4 and 0.67% TBA solution. The mixture was heated for 45 min at 100°C, and then n-butanol was added. The absorbance of the supernatant was measured at 535 and 520 nm, and compared to a standard value.

Hepatic level of protein carbonyl content was determined using the DNPH reaction, according to the previously described method (Levine et al., 1994). Briefly 200 μl of the hepatic homogenate in cold phosphate buffer (50 mM, pH 6.7, containing 1 mM EDTA) was mixed with 800 μl of DNPH (10 mM dissolved in 2.5 M HCl). The sample was incubated in the dark at room temperature for 1 h with vortexing every 15 min followed by the sequential addition of 1 ml of 20% TCA and 10% TCA. After centrifugation at 10,000 × g for 10 min at 4°C, a pellet was obtained and resolved sequential in 1 ml of an ethanol/ethyl acetate mixture (1:1, v/v) and in 500 μl of guanidine hydrochloride. The absorbance of supernatants (200 ml) was measured at 370 nm using a spectrophotometer (Molecular Device Corp).

### Analysis of Pro- and Anti-Inflammatory Cytokines in the Hepatic Tissues

Liver tissues were homogenized with RIPA buffer and centrifuged at 10,000 × g for 15 min at 4°C. The supernatant fraction was used for determination of the protein levels of Interleukin-1β (IL-1β), tumor necrosis factor-α (TNF-α), and Interleukin 10 (IL-10) using commercial ELISA kits (Minneapolis, MN for IL-1β, BioSource, San Jose for TNF-α, and IL-10), following the manufacturer’s protocols.

### Western Blot Analysis for TLR4, CD14, and LBP

The protein of the hepatic tissues was extracted using RIPA buffer. Each sample was separated by 10% polyacrylamide gel electrophoresis and transferred to polyvinylidene fluoride membranes. After blocking in 5% skim milk, the membranes were probed overnight at 4°C with primary antibodies against toll-like receptor 4 (TLR4, Novus Biologicals Littleton), CD14 (Novus Biologicals Littleton), LPS-binding protein (LBP, Abcam Massachusetts), and pan actin (Novus Biologicals Littleton), followed by incubation for 2 h with HRP-conjugated secondary antibody. Western blots were visualized using an enhanced chemiluminescence advanced kit, and then semi-quantified using ImageJ (NIH).

### Histopathology in the Hepatic Tissues

For the histomorphological evaluation of liver injury, a portion of liver tissue was fixed in Bouin’s solution from six groups (n = 8). The paraffin-embedded liver was sectioned (4-μm thickness), and hematoxylin and eosin staining were performed. A pathologist examined the representative histopathological features such as necrosis or inflammatory cell infiltration and scored its relative severity light microscope.

### Immunohistochemistry for Neutrophil and IL-1β in the Hepatic Tissues

To investigate the infiltration of neutrophil and production of IL-1β in the hepatic tissues, another experiment (each three mice of four groups for naïve, control, CGplus 200 mg/kg and DDB mg/kg) were set. After same experiment schedule of drug treatment and stress exposure, hepatic perfusion and frozen section (9 um diameter) were conducted for immunohistochemistry. The monoclonal antibody (both 1:250, eBioscience) against neutrophil and IL-1β and secondary goat anti-rabbit IgG-HRP antibody (1:1000, Thermo Fisher Scientific) were used, and the positive signals were observed in florescence microscopy.

### Statistical Analysis

All data are expressed as the mean ± standard deviation (SD). The statistically significant differences between the groups were analyzed using one-way analysis of variance (ANOVA) followed by an unpaired Student’s t-test. Differences were considered statistically significant at *P* < 0.05 or *P* < 0.01.

## Results

### Fingerprint of CG^plus^

The six main chemical compositions in CG^plus^ were confirmed using HPLC analysis as follows; scopoletin (retention time 12.7 min) in A. gmelinii, quercitrin (8.8 min) and quercetin dehydrate (16.8 min) in W. villosa var. xanthioides, and rosmarinic acid (13.6 min), salvianolic acid B (14.8 min), and tanshinone IIA (37.2 min) in *S. miltiorrhiza*, respectively (**Figures 1A, B**). In semi-quantitative analysis, quercitrin (16.16 μg/g) was most abundant while tanshinone IIA was fewest (0.46 μg/g) in CG^plus^ (data not shown).

### Effect on the Serum Corticosterone and Adrenaline Levels

**Figure 2:**
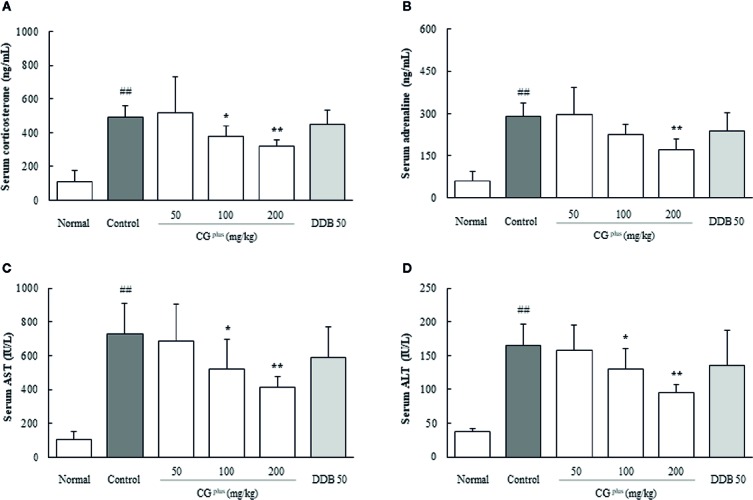
Serum levels of stress hormones and hepatic enzymes. Mice were orally dosed with CG^plus^ daily for 5 days and then subjected to immobilization stress for 6 h. Serum levels of corticosterone **(A)**, adrenaline **(B)**, AST **(C)**, and ALT **(D)** were then determined using an ELISA or Auto Chemistry Analyzer. Data are expressed as the means ± SD (*n* = 8). ^##^ *P* < 0.01 compared with the naive group; *P < 0.05, **P < 0.01, compared with the control group.

Immobilization stress drastically elevated the serum levels of corticosterone and adrenaline approximately 4.7- and 5.4-fold, respectively, compared to the naive group. Pretreatment with CG^plus^ significantly attenuated these abnormal increases in both the serum corticosterone level (*P<* 0.05 for 100 mg/kg, and 0.01 for 200 mg/kg) and adrenaline (*P<* 0.05 for 200 mg/kg) compared to the control group (**Figures 2A, B**). DDB pretreatment did not significantly affect the change in corticosterone or adrenaline level.

### Effect on the Serum AST and ALT Levels

Immobilization stress markedly increased the serum levels of both AST and ALT by approximately 7.1-and 4.5-fold, respectively, compared to the naive group, whereas these alterations were significantly attenuated by pretreatment with CG^plus^ compared to the control group (*P<* 0.05 or 0.01 for 100 and 200 mg/kg, **Figures 2C, D**). Pre-administration with DDB (50 mg/kg) did not significantly affect the serum liver enzymes.

### Histopathologic Findings in the Hepatic Tissues

**Figure 3:**
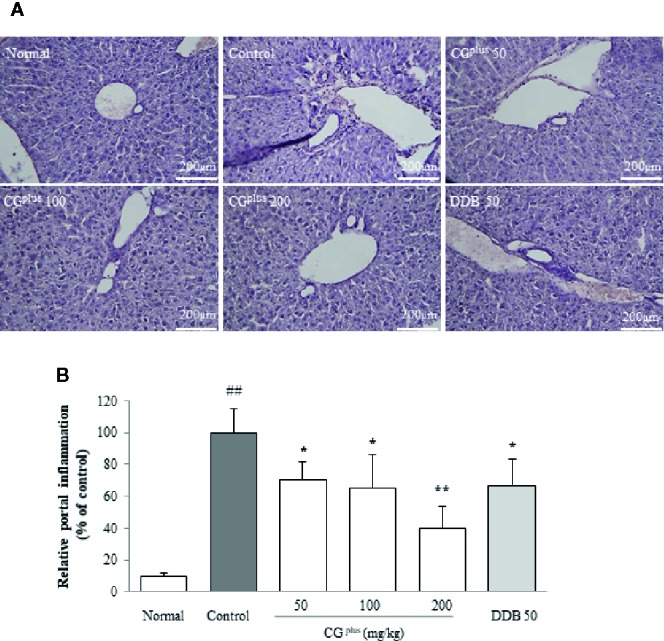
Histopathologic findings in hepatic tissues. Mice were orally dosed with CG^plus^ daily for 5 days and then subjected to immobilization stress for 6 h. The paraffin-embedded liver tissues were sectioned (4-μm thickness), and hematoxylin and eosin staining was performed. The representative histopathological features were examined under light microscope [**(A)**, 200× magnification], and the gross inflammations in portal area were assessed **(B)**. Data are expressed as the means ± SD (*n* = 8). ^##^ *P* < 0.01 compared with the naive group; *P < 0.05, **P < 0.01, compared with the control group.

Immobilization stress notably induced the typical inflammatory features including local necrosis and inflammatory cell infiltration especially around centrilobular region of hepatic tissues. These pathologic findings were considerably tempered by pretreatment with CG^plus^ or DDB (**Figures 3A, B**).

### Effect on ROS and NO in the Hepatic Tissues

**Figure 4:**
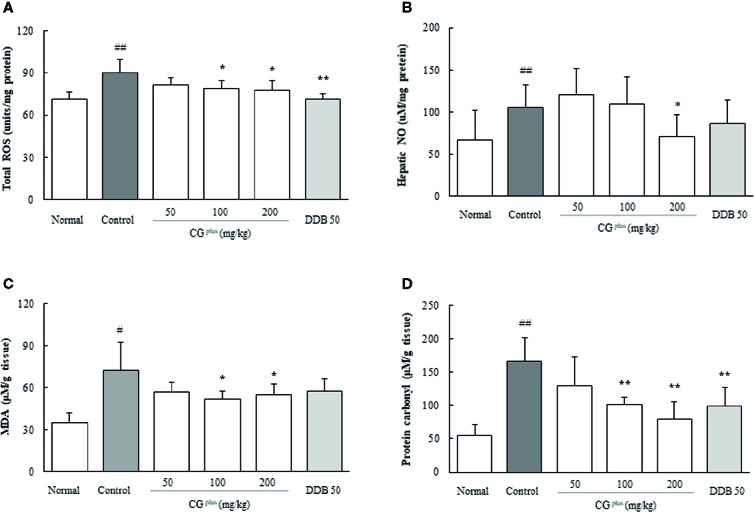
Hepatic levels of oxidative stress parameters. Mice were orally dosed with CG^plus^ daily for 5 days and then subjected to immobilization stress for 6 h. The hepatic levels of ROS **(A)**, NO **(B)**, MDS **(C)**, and protein carbonyl **(D)** were measured. Data are expressed as the means ± SD (*n* = 8). ^#^ *P* < 0.05, ^##^ *P* < 0.01 compared with the naive group; *P < 0.05, **P < 0.01, compared with the control group.

Immobilization stress significantly increased the hepatic levels of both ROS (1.2- fold) and NO (1.6-fold) compared to the naive group, whereas CG^plus^ pretreatment significantly attenuated those alterations of ROS (*P<* 0.05 for 100 and 200 mg/kg, **Figure 4A**) and NO (*P<* 0.05 for 200 mg/kg, **Figure 4B**) compared to the control group. Pre-administration with DDB (50 mg/kg) significantly ameliorated the over production of ROS in hepatic tissues.

### Effect on MDA and Protein Carbonyl in the Hepatic Tissues

Immobilization stress markedly increased the hepatic levels of both MDA and protein carbonyl by approximately 2.2- and 3.1-fold, respectively, compared to the naive group. Whereas these alterations were significantly attenuated by pretreatment with CG^plus^ (100 and 200 mg/kg) compared to the control group (*P<* 0.05 for MDA and 0.01 for protein carbonyl, **Figures 4C, D**). Pre-administration with DDB (50 mg/kg) also significantly ameliorated the increase of protein carbonyl levels.

### Effects on Pro- and Anti-Inflammatory Cytokines in the Hepatic Tissues

**Figure 5:**
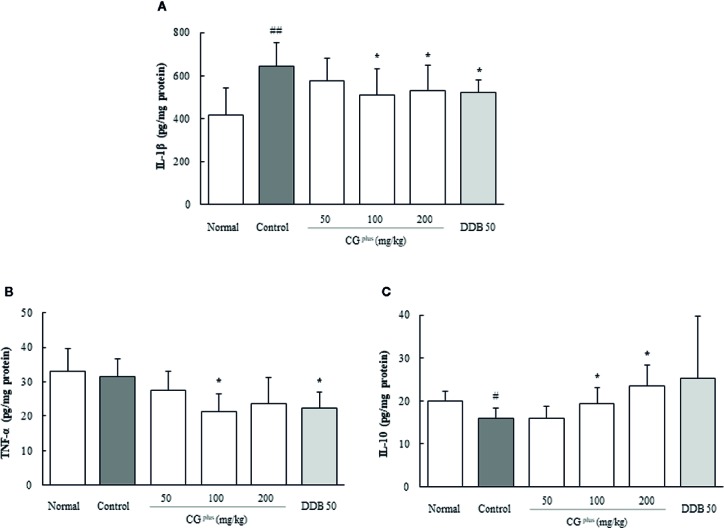
Hepatic levels of cytokines. Mice were orally dosed with CG^plus^ daily for 5 days and then subjected to immobilization stress for 6 h. The hepatic levels of IL-1β **(A)**, IL-10 **(B)**, and TNF-α **(C)** were determined using an ELISA. Data are expressed as the means ± SD (*n* = 8). ^#^ *P* < 0.05, ^##^ *P* < 0.01 compared with the naive group; *P < 0.05 compared with the control group.

Immobilization stress considerably increased the hepatic IL-1β level by 1.6-fold but lowered the IL-10 level by 0.8-fold compared to the naive group, whereas CG^plus^ pretreatment significantly attenuated those alterations compared to the control group (*P<* 0.05 for 100 and 200 mg/kg in both IL-1β and IL-10, respectively, **Figures 5A, B**). The serum TNF-α level was not changed by immobilization stress, but it was lowered significantly by pretreatment with CG^plus^ compared to the control group (*P<* 0.05 for 200 mg/kg, **Figure 5C**). Pre-treatment with DDB significantly affected only the serum TNF-α level compared to the control group (*P<* 0.05).

### Immunohistochemistry for Neutrophil and IL-1β in the Hepatic Tissues

**Figure 6:**
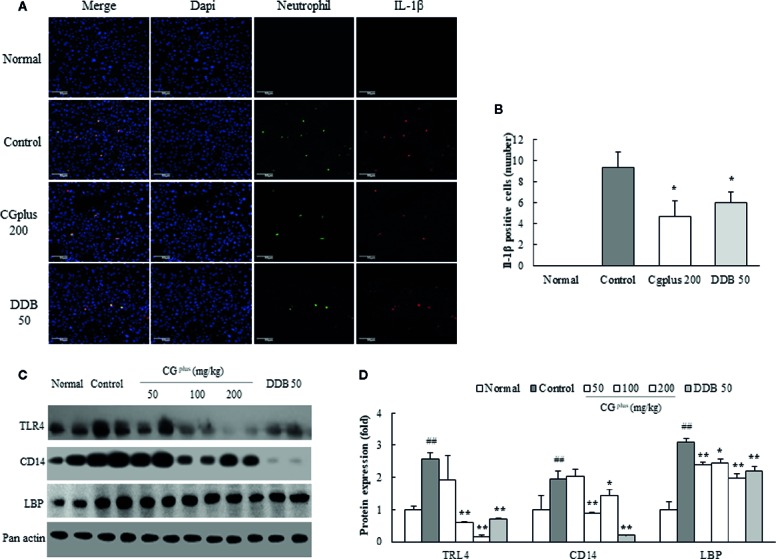
Immunohistochemistry for IL-1β positive neutrophil and protein analysis for LPS-receptor. Mice were orally dosed with CG^plus^ daily for 5 days and then subjected to immobilization stress for 6 h. After hepatic perfusion and frozen section, the immunohistochemistry staining was conducted against neutrophil and IL-1β (*n* = 3). Their signals (green or red color) observed under florescence microscopy **(A)** and quantified **(B)**. Western blot analyses were performed to measure the protein levels of TLR-4, CD14, and LBP [*n* = 8, **(C)**] and quantified **(D)**. Data are expressed as the means ± SD (*n* = 3 or 8). ^#^ *P* < 0.05, ^##^ *P* < 0.01 compared with the naive group; *P < 0.05 compared with the control group.

Immobilization stress notably induced the infiltration of neutrophils and production of IL-1β in hepatic tissue, which were significantly attenuated by pretreatment with 200 mg/kg of CG^plus^ or DDB (*P<* 0.05, **Figures 6A, B**).

### Effects on Activities of TLR4, CD14, and LBP in the Hepatic Tissues

Immobilization stress notably increased the protein activities of hepatic TLR4, CD14, and LBP compared to the control group, whereas these alterations were significantly attenuated by pretreatment with CG^plus^. The hepatic CD14 protein level was drastically reduced by pretreatment with DDB (**Figures 6C, D**).

## Discussion

The stress coping response is generally carried out *via* the hypothalamic-pituitary-adrenal (HPA) axis and sympathetic-adrenomedullary (SAM) system, which leads to the secretion of “stress hormones,” including corticosterone and adrenaline (Tsigos and Chrousos et al., 2002; Ulrich-Lai and Herman, 2009). We adapted an immobilization stress model, which drastically elevated the serum levels of stress-induced corticosterone and adrenaline by approximately 5-fold (**Figures 2A, B**). These stress hormones are known to alter the hemodynamics (hepatic hypoxia) and actions of immune cells, leading to an accelerated hepatic inflammatory response (Swain, 2000; Chida et al., 2006).

As was expected, immobilization stress induced acute liver injury as demonstrated by the rapid elevation of serum AST (7.1- fold) and ALT (4.5-fold) (**Figures 2C, D**). The elevations of the stress hormones and abnormal release of hepatic enzymes were significantly attenuated by pretreatment with CG^plus^. These results were supported by histopathologic findings including centrilobular necrosis which was attenuated by CG^plus^ treatment (**Figure 3**). Stress situation is known to reduce the hepatic blood flow, which is mediated by hepatic sympathetic nerve *via* secretion of norepinephrine at nerve terminal (Chida et al., 2005). This hepatic hypoxia causes the production of ROS in mainly mitochondria, leading to endoplasmic reticulum stress (Xu, 2005), and then kupffer cells and endothelial cells are activated to produce ROS and secrete various inflammatory cytokines under reperfusion status (Carden and Granger, 2000; Teoh and Farrell, 2003). As expected, hepatic levels of ROS and NO as well as the oxidative products (MDA and protein carbonyl) were notably increased by immobilization stress, whereas these alterations were significantly attenuated by pretreatment with CG^plus^ (**Figures 4A–D**).

The above hepato-protective effects of CG^plus^ under stress condition were supported by the normalizing action on the opposite deviation of IL-1β and IL-10 in hepatic tissue (**Figures 5A, B**). These two cytokines are well recognized as typical pro- and anti-inflammatory cytokines (Cavaillon, 2001), and the imbalance between pro- and anti-inflammatory cytokines determines the infiltration of immune cell and the resident cell functions in hepatic tissue (Sun and Ran, 2004). Intriguingly, the hepatic level of TNF-α, another typical pro-inflammatory cytokine, was not increased in our stress model (**Figure 5C**). One group showed strong evidence for the critical role of IL-1β in liver injury in an immobilization stress model (Tseilikman et al., 2012), whereas another group presented a null effect of TNF-α on hepatic damage using electric foot-shock stress (Chida et al., 2004a).

Above findings were supported by immunochemistry against hepatic infiltration of neutrophils and IL-1β. Hepatic infiltration of neutrophils is known well as an acute response to liver injury, such as drug/chemical-related hepatic inflammation and ischemia-reperfusion injury (Ramaiah and Jaeschke, 2007). IL-1β is primarily produced by cells of the monocytic lineage (Eder, 2009), and then our results showing the exact location of IL-1 β on neutrophils suggests the neutrophil-derived production of it (**Figures 6A, B**). In addition, stress hormones have been known to alter the intestinal permeability, resulting in an overinflux of gut-derived LPS into the hepatic portal vein, which is a potential cause of stress-associated hepatic injury (Saunders et al., 1994; Vanuytsel et al., 2014). In our study, immobilization stress induced the remarkable activation of TLR4 (a receptor for LPS) and its cofactor (CD13) as well as LBP (LPS-binding protein carrying LPS to TLR4) moderately in hepatic tissue, which was significantly attenuated by pretreatment with CG^plus^ (**Figures 6C, D**). We however don’t have the direct evidence for the changes of serum LPS concentration and intestinal permeability, which cannot exclude completely other factors besides LPS influx from intestine. In fact. there are additional possibilities that TLR4 would be activated by other factors such as damage-associated molecular patterns (DAMPs) (Lee and Seong, 2009). These findings should be further investigated along with the mechanisms of stress-induced haptic injury and evaluated for clinical relevance in the future. We used DDB, a known hepatoprotective agent (El-Beshbishy, 2005), as a reference drug in our experiment. In accordance with our present experimental model, DDB showed the antiapoptotic and immune-modulating effects on hepatic ischemia-reperfusion injury (El-Bahy et al., 2011) and LPS-derived immunological liver injury (Liu et al., 2012). DDB (50 mg/kg) was only partially positive and was less effective than CG^plus^.

CG^plus^ is a water extract of three medicinal plant mixtures that treats the main etiologies of liver disorders “*blood stasis*,” “*stagnation of vital energy*,” and “*dampness and Phlegm*” according to the Traditional Chinese/Korean medicine theory. We may need to examine other extraction methods producing the higher pharmacological efficacy than water extract. A. gmelinii showed the synergistic effects with *Curcuma longa* L. on nonalcoholic steatohepatitis (Kim et al., 2017). Hepatoprotective effect of W. villosa var. xanthioides was also reported from a chronic liver injury model (Wang et al., 2013). Furthermore, *S. miltiorrhiza* showed the neuroprotective actions against the psychological stress (Kim et al., 2015). Quercitrin, a main chemical compound in CG^plus^ was known to modulate macrophage activity by inhibition of peroxynitrite production (Kim et al., 2007). Silymarin exerted the hepatoprotective action in cute stress model (Kim et al., 2016) while rosmarinic acid pharmacological actions of hepatoprotection and anti-Tau aggregation in a strepotozotocin-induced diabetic and chronic restraint stress animal models (Mushtaq et al., 2015; Shan et al., 2016). Another compound, Tanshinone IIA also inhibited oxidative stress and apoptosis in a fatty liver model (Yang et al., 2017). The present results evidenced that an herbal combination composed of A. gmelinii, W. villosa var. xanthioides, and *S. miltiorrhiza* protected the liver from acute stress-derived inflammation. Although it was conducted as a different animal model with the present study, CG^plus^ showed the synergistic effects against CCl_4_-induced hepatotoxicity (Lee et al., 2019). Our current experimental design however cannot explore the synergistic action of above three plant and their active compounds corresponding for the pharmacological actions. We will consider the issue including their influence on hepatic blood flow and the quantitative level of LPS influx into liver using the further studies in the future. None specific adverse effects of CP^plus^ or DDB were observed in experimental process.

Taken together, our study showed an evidence of CG^plus^ protecting the liver against stress-derived injury, and its underlying mechanisms involved the regulation of pro- (IL-1β) and anti-inflammatory (IL-10) cytokines. However, further studies are needed to determine the synergistic effects of the three herbs, the corresponding active compounds, their influence on hepatic blood flow and the quantitative level of LPS influx into liver.

## Data Availability Statement

The datasets generated for this study are available on request to the corresponding author.

## Ethics Statement

The animal study was reviewed and approved by Institutional Animal Care and Use Committee of Daejeon University (DJUARB2016-004).

## Author Contributions

H-GK and Y-HK both participated mainly in the design of the experiments and manuscript preparation. S-BL, J-SL, S-WC, and D-GK conducted the assays and analyses. C-GS supervised whole processes of experiments and manuscript preparation. All authors read and approved the final manuscript.

## Funding

This study was supported by a grant from the Korean Institute of Oriental Medicine (K16840) and the by the National Research Foundation of Korea (NRF), Republic of Korea (No. 2019R1A2C2088201).

## Conflict of Interest

The authors declare that the research was conducted in the absence of any commercial or financial relationships that could be construed as a potential conflict of interest.

## Abbreviations

ALT, alanine aminotransferase; AST, aspartate aminotransferase; DDB, dimethyl dimethoxy biphenyl dicarboxylate; HPA, hypothalamic-pituitary-adrenal; IL-1β, Interleukin-1β; IL-10, Interleukin-10; LBP, lipopolysaccharides-binding protein; LPS, lipopolysaccharides; NO, Nitro oxide; ROS, reactive oxygen species; SAM, sympathetic-adrenomedullary; TLR4, toll-like receptor 4; TNF-α, tumor necrosis factor-α.
