# Supplementary material for: An Herbal Formula CGplus Ameliorates Stress-Induced Hepatic Injury in a BALB/c Mouse Model
Source: Front Pharmacol. 2020 Apr 14;11:447. doi: 10.3389/fphar.2020.00447 (PMC7171450; doi:10.3389/fphar.2020.00447)
Supplement: Supplementary file 1 [file Table_1.pdf]

Supplementary table 1. Taxonomic names of three herbs in CG^{plus}

Herbal name	Full taxonomic names	Part used
Artemisiaiwayomogi	Artemisia gmelinii Weber ex Stechm.	Herba
Amomumxanthioides	Amomum villosum var. xanthioides (Wall. ex Baker) T.L.Wu&S.J.Chen	Fructus
Salvia miltiorrhiza	Salvia miltiorrhiza Bunge	Rhizome
